# A 3D-Planned Inward Fragmentation Technique for the Removal of Impacted Mandibular Third Molars: A Case Series

**DOI:** 10.3390/jcm13206098

**Published:** 2024-10-13

**Authors:** Wilfried Engelke, David Streit, Pablo Acuña-Mardones, Randal von Marttens, Víctor Beltrán

**Affiliations:** 1 Faculty of Medicine, Georg-August-University of Göttingen, 37075 Göttingen, Germany; wengelke@med.uni-goettingen.de; 2 Private Practice in dentaMEDIC, 97638 Mellrichstadt, Germany; david.streit@dentamedic.de; 3 Clinical Investigation and Dental Innovation Center (CIDIC), Dental School and Center for Translational Medicine (CEMT-BIOREN), Universidad de La Frontera, Temuco 4811230, Chile; r.vonmarttens01@ufromail.cl; 4 Program of Master in Dental Sciences, Dental School, Universidad de La Frontera, Temuco 4811230, Chile

**Keywords:** computer-assisted surgery, digital planning, 3D navigation, inward fragmentation, mandibular, third molar, bone preservation, endoscopy, inferior alveolar nerve

## Abstract

**Background/Objectives**: The extraction of impacted mandibular third molars (M3Ms) carries significant risks, especially regarding the inferior alveolar nerve (IAN). This study aimed to evaluate the effectiveness of a 3D-planned inward fragmentation technique (3Dp-IFT) to improve surgical outcomes, reduce complications, and preserve bone structure in cases involving complex M3M impactions. **Methods**: Twenty-three patients aged between 18 and 36 years requiring M3M removal were included. Preoperative planning involved the use of cone–beam computed tomography (CBCT) for precise localization of the furcation area, followed by the creation of a 3D navigation template using PlastyCAD software version 1.7. The surgical procedure was performed under local anesthesia, with meticulous endoscopic assistance to ensure accurate access and minimize trauma. Postoperative outcomes, such as bone loss, pain, swelling, and mouth opening range, were carefully measured. The data were systematically organized and analyzed descriptively using Microsoft Excel. **Results**: No disturbances to the IAN or lingual nerve were observed. The mean buccal bone loss was 2.2 mm, with a standard deviation of 1.2 mm. Postoperative pain and swelling were generally low, with significant reductions within the first week. The use of the 3D navigation template significantly improved surgical access, enhancing safety and minimizing complications. **Conclusions**: The 3Dp-IFT technique represents a significant advancement in the minimally invasive removal of M3M by allowing precise access to critical anatomical areas while minimizing bone loss and postoperative complications. This approach is particularly beneficial for complex cases involving M3M near the IAN, thereby improving surgical safety and patient outcomes.

## 1. Introduction

The removal of impacted mandibular third molars (M3Ms) is a common procedure in dentistry, especially among oral and maxillofacial surgeons [1,2]. Juoadzbalys and Daugela highlighted the frequent impaction of third molars, often linked to varying surgical difficulty and complications such as nerve damage [3]. Pathological conditions including infections, caries, cysts, or tissue damage are common indications for M3M removal [4,5]. Despite the frequency of this procedure, both intraoperative and postoperative complications remain prevalent [6], with inferior alveolar nerve (IAN) damage being the most concerning, occurring in 0.1 to 22% of cases in which the nerve is exposed [7,8,9].

Numerous studies have analyzed risk factors for extractions of the M3M and concluded that the degree of impaction, pre-existing infection, and pathology of the M3Ms have a significant impact on the occurrence of complications [10].

Radiographic diagnostics play a fundamental role in judging the indication of surgery as well as the outcome following removal of M3M. Compared to conventional panoramic radiographs, cone–beam computed tomography (CBCT) provides superior visualization of bone and positional relationships between the nerve canal and roots, thus facilitating analysis of the anatomical context. Consequently, the surgeon adjusts their surgical approach accordingly, thereby minimizing the development of complications [11,12].

To further reduce the morbidity rate after osteotomy of M3M, it is necessary to develop and apply minimally invasive methods that minimize intraoperative trauma and thus reduce postoperative complications. Adequate visualization of the surgical field is essential. In particular, the use of endoscopes and/or microscopes can meet requirements for a largely atraumatic and gentle approach in a difficult-to-access surgical field [1]. For instance, Choi combined endoscopic visualization with an occlusal exposure technique for removing partially impacted M3M. This approach reduced the need for large flap formations and unnecessary osteotomies, significantly decreasing the complication rate [13]. Furthermore, advancements in minimizing invasiveness and enabling quality control have been achieved through the introduction of computer-assisted surgery [14]. At the same time, diagnosis using CBCT images is now predominantly recognized and approved as a standard procedure in planning and navigation in implant surgery and prosthetics in combination with optical scanners, precise implant insertions, and adequate prosthetics without the formation of mucoperiosteal flaps [15]. Recently, several studies and case reports from the field of endodontics have demonstrated a 3D-planned and navigated approach for trepanation of anterior teeth with heavily obliterated canals [16,17,18]. These studies have consistently shown satisfactory results in terms of precision and feasibility. With appropriate indications, a digital workflow combining intraoral scans and CBCT data could become a routine practice in dentistry [18].

In addition, Qi et al. (2022) demonstrated that 3D-printed titanium guides improve accuracy and safety in the extraction of horizontally impacted lower third molars, although they extend the surgery time without increasing postoperative complications [19]. The advantages of CBCT imaging—combined with template-guided, endoscopically assisted minimally invasive surgery—provide maximum safety, thus reducing intraoperative trauma and postoperative complications.

The inward fragmentation technique (IFT) appears to be more advantageous than conventional methods, especially in terms of reducing pain, swelling, loss of alveolar bone height, and injuries to the lingual nerve following third molar removal. This technique also leads to better postoperative quality of life compared to conventional surgery, as well as a reduction in alveolar bone loss [20].

The main challenges in third molar extraction include anatomically complex cases, the risk of damage to the inferior alveolar nerve, alveolar bone loss, and postoperative complications such as pain and trismus. The use of minimally invasive techniques like 3Dp-IFT can mitigate these risks, although precise surgical planning, specialized equipment, and trained surgeons are required.

IFT allows surgeons to avoid extensive soft tissue detachment, while simultaneously reducing bone loss and intraoperative bleeding. This is achieved by focusing the preparation on the internal separation of the tooth, thus avoiding damage to nearby anatomical structures [21]. Building on this approach, the present study seeks to address the following question: does 3Dp-IFT improve surgical outcomes and preserve bone structure in patients with complex M3M impactions?

The aim of this study is to evaluate the effectiveness of a 3D-planned inward fragmentation technique (3Dp-IFT) in improving surgical outcomes, reducing complications, and preserving bone structure in complex M3M impactions.

The relevance of this research lies in the high incidence of complications during the extraction of impacted M3M [22,23,24]. The 3Dp-IFT technique represents a significant advancement in improving surgical precision and reducing risks, particularly in complex cases near the IAN.

## 2. Materials and Methods

### 2.1. Patients

A total of 23 patients (9 male, 14 female) aged between 18 and 36 years participated in the study. Each patient had only one M3M removed.

The inclusion criteria were as follows: healthy patients over 18 years old, without significant medical history, who showed a clinical or radiological indication for the extraction of a lower third molar close to the IAN in the panoramic radiograph. Patients who were uncooperative, lacked the capacity to consent, were pregnant, or had systemic diseases that could influence the evaluation of the surgical method were excluded.

This study was approved by the Scientific Ethics Committee of the Universitätsmedizin Göttingen, Göttingen, Germany (Code 16/12/15) and the Scientific Ethics Committee of the Universidad de La Frontera, Temuco, Chile, Folio Number 118/16.

**Figure 1:**
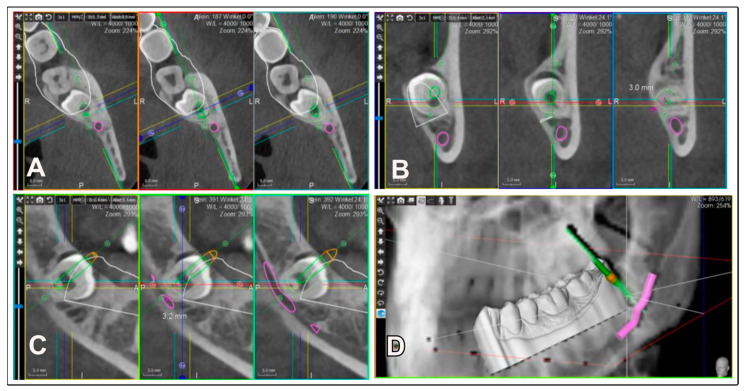
Three-dimensional planning of the surgical access for the IFT in CBCT. The target zone, i.e., the furcation area, is planned in three spatial planes: axial, coronal, and sagittal. (**A**): axial view, (**B**): coronal view, (**C**): sagittal view, (**D**): 3D representation of the direction and depth of the access to the furcation area.

The patients had a close spatial relationship between the third molars and the IAN, as observed on the panoramic radiograph, necessitating a precise diagnostic assessment with a CBCT (WhiteFox, Acteon, France) at 105 kV and 10 mA with a 12 × 8 cm field of view (FOV). The images were reconstructed with a voxel size of 0.15 mm using WhiteFox Control software version 2.11.1 (WhiteFox, Acteon, France). Based on this CBCT examination (Figure 1), a 3D plan for minimally invasive removal was carried out.

### 2.2. Digital Planning

Following the methodology described by Engelke et al. (2013), the aim of the digital planning was to accurately define the molar furcation for navigation [20]. After achieving the planning objective, the inward fragmentation technique was to be used for the removal of the molars.

**Figure 2:**
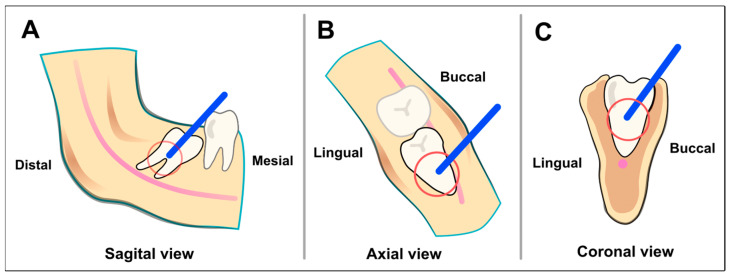
Schematic representation of the access drilling in the three spatial planes. The drill channel is aligned so that a surgical handpiece coming laterally from the cranial side directly reaches the function. The center of the red circle corresponds to the furcation.

A standard drilling tunnel was planned for access (Figure 2). The tunnel had a length of 11 mm to the hard tissue surface. It was oriented in a buccal–occlusal direction and ended at its deepest point at the furcation of the third molar or at the deepest point of the pulp.

The buccal angle, measured between the buccal surfaces of the first and second molars and the drilling direction, was to be between 20° and 25°, and the occlusal angle (between the occlusal surfaces of the molars and the direction of access) between 40° and 50°. A safety distance of 2 mm to the neighboring tooth, IAN, and lingual compacta was maintained.

Subsequently, an intraoral scan TRIOS (3Shape, Copenhagen, Denmark) of the affected area was made and, after virtual model creation, the superposition of intraoral information on hard and soft tissues obtained from surface scan with the CBCT image was also performed using WhiteFox Imaging software version 4.1 (WhiteFox, Acteon, France). Exact matching between the DICOM dataset and the STL format was achieved through automatic BestFit alignment after performing an initial alignment at any point in both formats. The optimal superposition was then verified in the cross-sectional and sagittal planes.

The virtual construction of the navigation template was carried out using the freeform software PlastyCAD version 1.7 (3Diemme, Italy), which was then produced using the SLA-based 3D printer DigitalWax 028D (DWS, Vicenza, Italy).

For the construction of the template, the following parameters were set: template sleeve diameter: 10 mm, inner diameter of the outer sleeve: 3.5 mm, inner diameter of the inner sleeve: 2.1 mm, depth marking: 20 mm (distance from sleeve end to furcation).

### 2.3. Surgical Procedure

All surgeries were performed by a surgeon with over 5 years of experience in oral microsurgery and specializing in computer-assisted techniques. The surgical intervention was carried out under local anesthesia (3 mL UDS, 4% Articaine with 1:200,000 Adrenaline) with two-nerve-block anesthesia of the IAN and the buccal nerve as well as a terminal anesthesia. The surgeon worked in a 12 o’clock position with a view of the endoscope screen. The support endoscope (30° forward-looking optics, 2.7 mm diameter, Karl-Storz, Tuttlingen, Germany) was always placed at the distal end of the surgical site.

**Figure 3:**
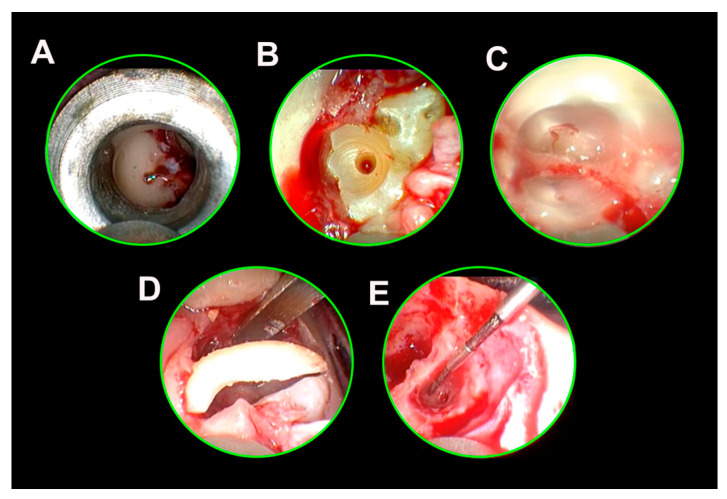
Process of the 3Dp-IFT. (**A**): Endoscopic view through the drill channel with the drill sleeve in place; (**B**): drilling after removal of the sleeve and expansion of the drill hole; (**C**): internal separation of the tooth; (**D**): inward fragmentation of the distal fragment; (**E**): situation after removal of the segmented tooth.

Initially, soft tissue preparation was carried out in the form of an occlusal mini-flap. Endoscopic visualization allowed the avoidance of a mucoperiosteal flap (Figure 3A).

After testing the navigation template and checking the fit, pilot drilling was carried out until the depth stop was reached (Figure 3B). This was then endoscopically controlled. The first 3 mm of the pilot drilling was expanded with a rose bur. The expansion drilling was then carried out up to the depth marking. This drilling was also visually inspected with an endoscope.

### 2.4. Expansion of Access to the Furcation

Following the expansion drilling, the separation of the tooth was then carried out buccally and lingually. In the buccal and middle third of the crown, this was achieved using rose drills, while in the lingual third, where the vulnerable structures are located, diamond ball cutters were used. The goal of the space-creating trepanation was to represent the furcation area in its three-dimensional extent.

### 2.5. Interradicular Separation

The separation of the M3M then followed, starting from the furcation, preferably in a bucco-lingual direction (Figure 3C), always under direct endoscopic vision for assistance. The goal of the sectioning was to create an internal space to be able to apply the IFT.

The bone fragments were subsequently removed under endoscopic vision (Figure 3D,E).

**Figure 4:**
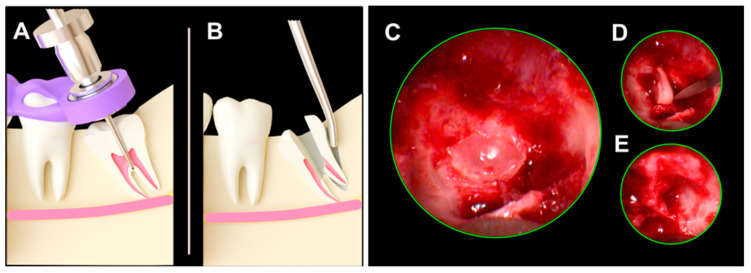
Three-dimensional navigation template for the pilot drilling. (**A**): The template is placed on the adjacent teeth, and the drill sleeve guides the pilot drill to the target position in the area of the function. (**B**): After performing the internal separation, the tooth fragments are mobilized inward and removed individually. (**C**): Residual root remnant following IFT. (**D**): Endoscopically assisted luxation of an apical root remnant in the tooth 4.8 post-extraction site. (**E**): Endoscopic inspection of the post-extraction site following root remnant removal.

The use of the guiding template for access to the furcation area and subsequent inward fragmentation are summarized in Figure 4. Note that the 3D navigation is used for the location to the critical zone for the internal fragmentation (Figure 4A), while the process of separation and tooth removal (Figure 4B) is performed without 3D guidance under endoscopic microsurgical conditions.

### 2.6. Determination of Bone Loss

**Figure 5:**
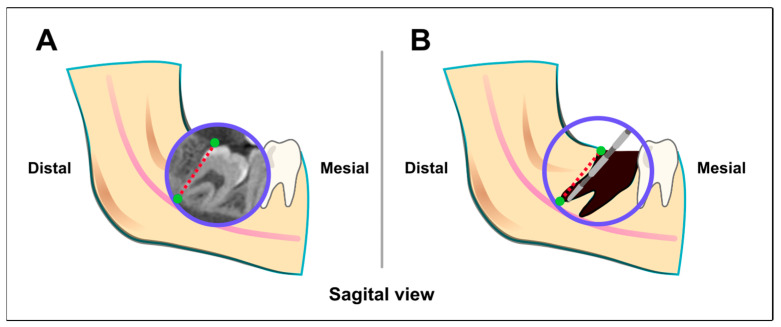
Determination of the buccal bone height in comparison. (**A**): Preoperative measurement of the bone height using CBCT image. (**B**): Direct measurement of the apex point (see Figure 3E) after removal of the tooth.

The bone height was measured using CBCT images prior to surgery, utilizing the measurement tool in WhiteFox 3D Diagnosys software version 4.2. The distance between the apex point of the distal root and the buccal bone border was measured (Figure 5A).

After the completion of fragment removal, the socket was thoroughly rinsed and visually inspected. Furthermore, the integrity of the thin bone walls was ensured. Finally, the buccal socket height was measured using special periodontal probes, and intraoperative bone height after M3M removal was documented (Figure 5B).

In justified individual cases, a new CBCT image was taken postoperatively. With this, the bone loss could be immediately graphically represented.

### 2.7. Statistical Analysis

The descriptive analysis of the data was performed using Microsoft Excel 2016 (Microsoft Corporation, Redmond, WA, USA). The mean, standard deviation, and median were calculated.

## 3. Results

### 3.1. Bone Loss Data

Table 1 provides information about bone loss. The buccal bone loss measured during each intervention, between the deepest point of the socket and the buccal bone edge (Figure 5), compared pre- and postoperatively, is presented in Table 1.

### 3.2. Postoperative Findings

The range of mouth opening (24 h post surgery) varied between 18 mm and 55 mm (mean: 29 mm), and after seven days, it ranged from 27 mm to 61 mm (mean: 50 mm). Pain perception was assessed using a VAS scale from 1–10. After 24 h, the median was 3.8 (range 1–8), and after one week, it was between 1 and 2 (median: 1.2). The swelling was graded on a scale of 0 (none) to 3 (severe swelling). On the first postoperative day, 17 patients had Grade 1 swelling, three patients had Grade 2, and one patient had Grade 3. After 7 days, one patient showed Grade 1, and all others were at Grade 0.

No adverse events were reported during the study. Complications such as instrument deviation, fracture, or misalignment of the template were not observed. The template was checked intraoperatively to ensure proper alignment.

## 4. Discussion

The removal of horizontally displaced M3M with large root branches is often performed by separating the roots and parts of the crown [25]. Kim et al. (2011) found that flapless access is regularly possible when the distal surface of the crown is completely in front of the ascending branch of the lower jaw. In these cases, the occlusal surface of the affected tooth is at nearly the same height as the occlusal plane of the second molar. Although flapless removal is desirable, it cannot often be found in complex positions of the tooth, especially near nerves. In such cases, the IFT method was introduced [26].

The development of the minimally invasive IFT more than 10 years ago [20] has proven especially effective for highly complex displacements with a strongly lingual or deeply impacted third molar. It must be emphasized that the use of support endoscopy is mandatory for IFT, although parts of the technique may be assisted by using loupes or surgical microscopes.

**Figure 6:**
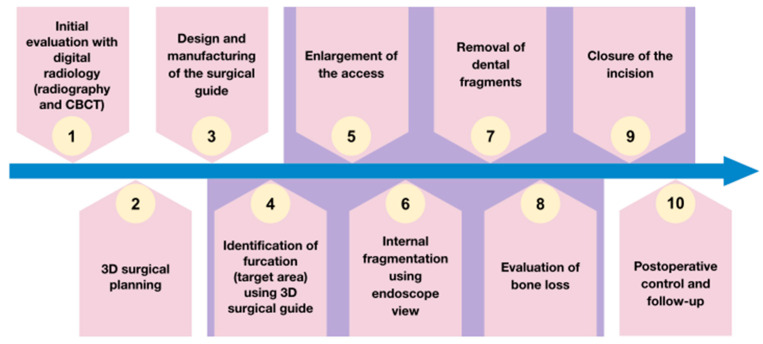
Workflow of 3Dp-IFT. Surgical stage (purple background).

The 3D-planned inward fragmentation technique (3Dp-IFT) presented in our present study was designed to evaluate if the critical target zone for division of the M3M, which is the furcation area, could be identified. This would facilitate the endoscopically assisted division of the M3M (Figure 6). Observations during microsurgery revealed that pilot drilling was helpful for orientation in complicated anatomical situations. The 3D-planned target drilling demonstrated high accuracy in identifying the division zone, which then facilitated rapid transverse root division. In particular, surgeons in the early stages of their learning curve in microsurgery significantly benefit from rapid anatomical orientation and a high level of surgical safety.

The identification of the furcation area in the case of single rooted M3M has been taken as a valuable indicator for later division.

It should be stressed that static 3D navigation as described in this article should always be accompanied by endoscopically assisted division of the furcation area.

Using continuous intermittent rinsing, a clear view to the division sone can be obtained.

Endoscopy is used in oral surgery to improve visualization of the surgical field in difficult access and thus contributes to reducing invasiveness [27]. For the application, a 30° forward-looking optic is introduced into the surgical field. With continuous irrigation, this serves to observe and visualize the anatomical structures [28]. As Farish describes, new surgical techniques combined with intensive training and sufficient experience have led to a development in dental surgery that has made the removal of third molars possible in a minimally invasive and less traumatic way [29]. Examples of these new techniques include the coronectomy method [30], the odontosection technique [31], or microsurgical removal via an occlusal access using visual aids [13].

In other disciplines of dentistry, such as implantology, the combination of 3D imaging X-ray procedures with intraoral scans and digital planning is already a standard procedure for quality assurance and simplification of processes [15]. Additionally, in endodontics, this is the latest state clinical studies have shown the possibility of transferring digital plans to the surgical area [16,17,18].

Emery et al. (2017) examined the results with a new technology, dynamic navigation technology, in which the instrument (e.g., handpiece) is manually guided freely on a screen at the patient under online navigation. This eliminates the need for a navigation template, which is statically positioned. The authors saw potential advantages through (1) improved visualization of critical structures, (2) better intraoperative control, (3) smaller access size, (4) suitability for practice, (5) shortened operation time, and (6) application in teaching. Possible limitations in the use of in-office dynamic navigation include increased costs, more time for preoperative planning, initial learning curve, and disturbances of the optical array by the surgeon or assistant during surgery [32].

Comparing these points with our procedure of static navigation template in combination with intraoperative support endoscopy, the following observations emerge.

The visualization of critical structures is given during the entire operation time in dynamic navigation, not in static, which has completed this in planning. However, the current situation of the surgical procedure can be checked at any time with the support immersion endoscope in case errors are suspected in the preparation. This also applies to intraoperative control, which is visually ensured. If positioning errors of the template are suspected, endoscopic control can provide certainty whether endangered structures are visible around the target point.

The precision of instrument guidance under dynamic navigation and instrument guidance with static template-guided navigation are therefore of the same magnitude for planning. Intraoperatively, template-guided navigation has the advantage that no optical systems must be fixed on the instrument and the patient; thus, the surgeon’s freedom of action is enhanced. Templates used in static navigation present certain disadvantages in terms of access size due to the lateral direction required when using a handpiece. However, this can be compensated by employing an angled piece and vertical access. In terms of suitability for practice, both systems perform well, but static navigation aligns more closely with the existing standard.

In dynamic navigation, when positioning references on the patient, one should expect restrictions in the operator’s field of movement and a risk of dislocation during the procedure.

Regarding operation time, only anecdotal data are available to us. As data were not collected, the learning curve of the operator in static navigation is lower than the curve of the dynamic system; however, the application of a support endoscope after IFT is also associated with a learning curve. Application for training in oral surgery is unrestrictedly possible, for both systems.

There are challenges in designing portable guides to improve the accuracy of impacted mandibular third molar extraction, as well as in the prior training of dental surgeons to perform these techniques. In this regard, a recent in vitro study demonstrated that, with the digital guide technique, tooth sectioning is more predictable and accurate, and the success of the operation is achievable with varying levels of proficiency among dental surgeons [33]. This technique can reduce the difficulty of tooth extraction and decrease the risk of damage to the surrounding soft and hard tissues, particularly to the IAN. In this context, our study further complements the clinical application by incorporating intraoperative endoscopic visualization during root sectioning, providing considerable advantages in the execution of the IFT technique steps.

In relation to the above, in Figure 4C–E, the advantage of intraoperative endoscopic visualization after root sectioning using a 3D guide can be observed, as it allows for controlled luxation and removal of apical root fragments with micro-instruments, which may be in direct contact with the IAN. In our experience, when such clinical situations arise, it is first possible to attempt luxation with micro-instruments under direct intraoperative magnification, and if that is not feasible, a small osteotomy around the root fragment can eventually be performed using piezosurgery. However, the advantage of intraoperative magnification control ensures that these maneuvers are precise and carefully controlled.

It should be added that intraoperative endoscopic control is not mandatory in static template-guided navigation, and under favorable conditions, microsurgical procedures can also be carried out with loupes or a microscope. With appropriately trained operators, this alternative will shorten the learning curve.

As already mentioned, the surgical removal of M3M is always associated with a certain reduction of the alveolar bone to obtain sufficient access to the surgical field. With the conventional buccal approach, the bone level can subsequently lie below the cement–enamel junction, leading to increased fragility of the lower jaw in this area [34]. Therefore, the degree of impaction of the tooth carries a high risk of postoperative complications such as inflammation, pain, and swelling [6,35].

To keep the loss of alveolar height as low as possible, the type and execution of the surgical procedure must be based on minimally invasive principles.

Thus, the nutrition of the tissue was maintained, and the preservation of the jaw ridge was ensured [13]. In addition, the extent of osteotomy was limited to the occlusal part, and lateral bone removal was avoided. The data show that the loss of bone substance was low despite the deep impaction and lingual displacement and was only due to the occlusal osteotomy used to expose the occlusal surface of the third molar. By referring back to the preoperative CBCT image of the postoperative bone surface, the bone loss could be realistically represented and accurately measured.

In the literature search, the author could find no studies dealing with metric analyses of bone loss in the removal of M3M. In the patient case shown, an exact volumetric evaluation could thus be presented for the first time. This showed that the 3D-planned, template-navigated, and endoscopically supported removal of M3M, especially in complex cases, can be a method of preserving bone substance and thus minimizing the risk of fractures and further complications.

An important question of this study concerned the resulting buccal bone loss compared to the previously described IFT without the use of a navigation template. The navigated procedure was compared with the results of the endoscopically assisted IFT [36]. It was found that the vertical bone loss in the current study with template-supported navigation was slightly greater than in the IFT study (0.8 mm). This can be explained by the pilot drilling directly in the anterior–lateral direction. The maximum buccal bone loss of 5.6 mm represents an outlier, possibly linked to the complexity of the case in terms of proximity to the inferior alveolar nerve and angulation. This value is significantly higher than the reported average of 2.2 mm, and it is suggested that further studies investigate these outlier cases in greater depth.

Compared to a more occlusal approach of the IFT without a navigation template, a certain narrow-defined reduction of bone height must already be accepted in the pilot drilling and its expansion. If, on the other hand, preparation is carried out exclusively under endoscopic control from occlusal, the lateral wall can be spared even more. For this, the use of an angled piece for the preparation of the access and the subsequent separation of the tooth is necessary.

However, surgical routines generally use straight handpieces, which are suitable for bone removal buccally but cannot be guided appropriately vertically from the occlusal perspective. The navigated IFT must therefore adapt more to the concept of the technique used in dental preparation to enable optimal bone preservation. This convergence of endodontic and surgical preparation techniques highlights that the more precise the planning and the better the cutting properties of the instruments used, the less bone loss can be expected with optimal planning through a vertical approach.

In contrast to the previously described IFT, the planning effort and, consequently, the treatment costs of the navigated procedure are significantly higher than those of the exclusively endoscopically assisted IFT. Therefore, in each individual case, the question arises as to whether the planning effort involved in the navigated template-supported technique is justified.

Regarding the operation time, it can generally be said that the template-supported technique shortens the phase of minimally invasive separation and inward fragmentation. Safe statements to this effect can, however, only be made when the procedure is used by sufficiently trained surgeons in routine practice. The tendency to shorten the operation by applying navigated planning was observed in the duration of the 3Dp-IFT; however, this must be substantiated by exact figures in subsequent studies.

IFT with 3D static navigation offers several advantages over traditional IFT. It allows for a safer preoperative analysis of the direction and depth of internal fragmentation, as well as more precise preparation in the furcation area, particularly in cases involving complex and deeply displaced M3Ms. Furthermore, the technique provides a well-defined area for endoscopically assisted preparation after the target zone is identified, while still allowing for the use of standard preparation techniques when exposing the furcation area.

On the downside, this approach leads to greater lateral bone loss than with conventional IFT, requires increased technical effort and higher treatment costs due to the need for a navigation template, and limits the availability of 3D information to the pilot phase of surgery. Despite these drawbacks, the enhanced precision and safety it offers, especially in complex cases, may justify the additional effort and cost in certain clinical scenarios.

Additionally, the disadvantages of guiding the drill channel from the anterolateral side when using a surgical handpiece may, in some cases, be compensated by using a contra-angle handpiece, provided that the degree of mouth opening allows for occlusal instrument guidance. This consideration can be evaluated during the planning phase and requires that the patient’s degree of mouth opening is taken into account to ensure successful implementation of the procedure.

Clinical anatomical studies will show whether this approach can be chosen in surgical routine as an alternative. Regardless, the bone loss of the lateral alveolar wall is comparatively small compared to classic buccal osteotomy.

The 3Dp-IFT is suitable particularly for those cases in which a complicated location of the M3M close to the IAN needs to be removed. This applies especially in the case of vertical displacement with a lingual inclination. Precise definition of the anatomical landmarks makes internal separation significantly easier provided that the endoscopically assisted IFT is applied.

3Dp-IFT will also significantly simplify the surgical process for the removal of displaced canines and retained upper third molars. The use of a 3D-planned guide to facilitate the removal of displaced teeth, in general, has been previously reported [34].

The initial cost of implementing the 3Dp-IFT technique varies and includes the acquisition of CBCT, 3D surgical planning software, 3D surgical guide design and manufacturing services, and endoscopic equipment. However, technological advancements and the increasing affordability of CBCT have made its use more widespread in contemporary dentistry. While 3D-planned surgery preparation saves time and enhances accuracy, it also raises overall treatment costs. These higher costs are typically justified in high-risk cases. Nevertheless, these initial expenses are offset in the long term by fewer complications and shorter recovery times, which ultimately reduce follow-up and postoperative treatment costs. It should be noted that a formal cost analysis was not conducted in this study.

This study was conducted on a specific group of patients who had access to advanced imaging technologies and computer-assisted surgery. As a result, the findings may not be fully applicable to settings where these resources are unavailable.

A key limitation of the study is the absence of a control group using conventional techniques. However, this was necessary to focus on a series of complex cases where the 3Dp-IFT technique provides distinct advantages in reducing complications. Future research should include a control group to enable more robust comparisons.

Additional limitations include the relatively small sample size and potential biases, such as the surgeon’s experience, misalignment of the guide splint, inadequate visibility of the surgical field, and the need for osteotomy in cases where internal fragmentation is incomplete. Moreover, the limited availability of 3D technology in some areas may restrict the generalizability of the results to other clinical settings.

The limitations of 3Dp-IFT include the need for specialized equipment and high-resolution imaging, the surgeon’s experience, and expertise in planning, which may increase initial costs. However, these limitations can be mitigated through broader adoption of this technology in clinics, as well as by implementing training programs in the technique, which would reduce costs in the long term.”

The results of this study have significant implications for clinical practice, as the 3Dp-IFT technique not only reduces the risk of postoperative complications but also improves long-term outcomes by preserving alveolar bone. Extensive bone loss can increase the risk of fractures of the alveolar socket and the mandible in complex cases of impacted third molars [37,38,39,40,41]. This is particularly relevant in younger patients, where bone preservation is crucial for long-term dental health.

The success of the 3Dp-IFT technique relies heavily on the surgeon’s experience and training in the use of computer-assisted technology. Implementing specific training programs for surgeons in the use of CBCT and 3D surgical planning is essential to ensure favorable clinical outcomes.

Originally, it could only be performed in high-complexity centers. The 3Dp-IFT technique could be adapted to different clinical settings through the use of low-cost 3D planning software and proper training for surgeons. With the expansion of access to CBCT, this technique could be implemented even in clinics with limited resources.

Finally, 3D planning of the target point itself is a new option for the training and further education of oral surgeons to virtually simulate the steps of the osteotomy in all details before a planned procedure to keep the risk of incorrect treatment in the subsequent operation as low as possible.

In each individual case, it must be decided whether the material and time expenditure of 3D planning is justified or whether internal separation can be carried out with sufficient safety through intraoperative endoscopic observation alone. This depends primarily on the learning curve of the surgeon.

In any case, the 3Dp-IFT is an important contribution to optimizing routine operations, improving current M3M techniques, and keeping bone loss during complex tooth removals to a minimum.

Future developments of the 3Dp-IFT technique include integration with augmented reality technologies and the 3D printing of personalized surgical guides, as well as the implementation of specialized training programs in the technique. These advancements will enable greater customization of surgical procedures, potentially further increasing precision and reducing the risk of complications, while adapting the technique to the specific needs of each patient.

## 5. Conclusions

The 3Dp-IFT technique is a further development of the existing technique for minimally invasive removal of M3M that aims to achieve precision access to the target area. This allows for internal separation of complexly displaced M3M with precise anatomical knowledge. As a result, the risk to surrounding structures is minimized.

Compared to IFT, this approach results in slightly greater bone loss on the buccal side of the mandible.

By effectively transferring preoperative planning to the intraoperative stage, IFT can be further optimized. This technique facilitates a minimally invasive approach to removing complex displaced teeth, thus minimizing associated risks.

The conclusions of this study are based on the results obtained from this case series, where no significant complications were observed in the use of the 3Dp-IFT technique. Nonetheless, a larger number of cases and controlled studies are required to fully validate these findings.

The 3Dp-IFT technique will have a potentially significant impact on clinical practice, particularly in reducing complications and improving surgical outcomes in cases of impacted mandibular third molars. Its ability to preserve alveolar bone and reduce the risk of nerve damage makes it a promising technique for managing these complex cases.

## Figures and Tables

**Table 1:** Buccal bone height before and after tooth removal.

	Preoperative Bone Height (mm)	Postoperative Bone Height (mm)	Bone Loss (mm)
Mean	16.5	14.3	2.2
Min	13.5	10	0.6
Max	20.1	18	5.6
SD	1.6	1.9	1.2

## Data Availability

The data presented in this study are available on request from the corresponding author due to legal reasons.
